# Birth Asphyxia: Risk Factors, Complications, and Outcomes in Neonates Admitted at a Tertiary Care Center in Gujarat, India

**DOI:** 10.7759/cureus.95271

**Published:** 2025-10-23

**Authors:** Hrishabh Soni, Nimisha Pandya, Prashant Soni, Shraddha Soni

**Affiliations:** 1 Paediatrics, Gujarat Medical Education and Research Society (GMERS) Medical College and General Hospital, Vadodara, IND; 2 Obstetrics and Gynaecology, Netaji Subhash Chandra Bose Medical College, Jabalpur, IND

**Keywords:** birth asphyxia, hypoxic-ischemic encephalopathy, maternal risk factors, neonatal mortality, perinatal asphyxia

## Abstract

Background

Birth asphyxia is a significant cause of neonatal morbidity and mortality, especially in low- and middle-income countries. It is often associated with long-term neurological and systemic complications. This study explores the incidence, maternal and neonatal risk factors, and outcomes of neonates admitted with birth asphyxia at a tertiary care center in Gujarat, India.

Objectives

This study aims to determine the incidence of birth asphyxia among NICU admissions, identify maternal and neonatal risk factors associated with birth asphyxia, and evaluate the immediate outcomes and complications in affected neonates.

Methods

This prospective observational study included 130 neonates diagnosed with birth asphyxia, defined by an Apgar score of ≤6 at 1 minute. Data were collected over 19 months from July 2022 to February 2024 at GMERS Medical College and General Hospital, Vadodara, Gujarat. Maternal and neonatal factors were analyzed using structured questionnaires and medical records. Descriptive and inferential statistics were applied to evaluate the data.

Results

Among 2,770 NICU admissions, 130 neonates (4.7%) were diagnosed with birth asphyxia. The majority of affected neonates were male (60.8%), and 79.2% of mothers belonged to a lower socioeconomic class. Key maternal risk factors included anemia (83.1%), hypertension (19.2%), and prolonged labor (32.3%). Hypoxic-ischemic encephalopathy (HIE) was observed in 72.3% of cases, with 10.8% presenting with Sarnat Grade III. Neonatal mortality was significantly higher in outborn cases (28.6%) compared to inborn cases (3.8%).

Conclusion

The study underscores the critical role of maternal health, socioeconomic factors, and timely neonatal interventions in reducing the burden of birth asphyxia. Improved antenatal care, robust intrapartum monitoring, and effective referral systems are essential to mitigate adverse outcomes. Further research and public health initiatives are needed to address the disparities in neonatal care.

## Introduction

Despite substantial advances in neonatal care, birth asphyxia persists as a significant global public health concern, particularly in resource-limited settings. It is a leading cause of neonatal mortality worldwide, contributing disproportionately to the burden in developing countries. Birth asphyxia, defined as the failure to initiate and sustain breathing at birth, is often associated with hypoxic-ischemic encephalopathy (HIE) and multi-organ dysfunction. These complications can result in long-term neurodevelopmental sequelae, severely affecting the quality of life of survivors and placing a substantial burden on families and healthcare systems [1].

In India, birth asphyxia accounts for approximately 20% of neonatal deaths, with substantial regional variations driven by socioeconomic and healthcare disparities [2]. Factors such as inadequate access to prenatal care, poor intrapartum monitoring, and delayed medical intervention exacerbate these outcomes [3]. Addressing this issue requires targeted strategies informed by robust data on incidence, risk factors, and outcomes. Comprehensive care models and community-based interventions are particularly important in addressing these challenges in resource-constrained settings [4].

This study was conducted to evaluate the incidence, maternal and neonatal risk factors, and clinical outcomes associated with birth asphyxia at a tertiary care center in Gujarat. By identifying modifiable risk factors and critical points of intervention, the study aims to provide actionable insights for improving perinatal and neonatal outcomes. The findings are expected to contribute to the formulation of evidence-based policies and practices to address this pressing health issue.

## Materials and methods

Study design and setting

This prospective observational study was conducted at the Neonatal Intensive Care Unit (NICU) of GMERS Medical College and General Hospital, Gotri, Vadodara, Gujarat. The study spanned 18 months, from August 2022 to February 2024, and employed a systematic approach to data collection and analysis. The first patient was recruited on 25/08/2022 following institutional ethics approval on 24/08/2022. The NICU manages a caseload comprising both inborn neonates and referred cases, serving as a referral center for outborn neonates [5], providing an ideal setting to study the multifaceted nature of birth asphyxia.

Study population

The study population included neonates with an Apgar score of ≤6 at 1 minute of birth, born at ≥37 weeks of gestation, and admitted to the NICU during the study period [6]. Neonates with anesthesia-related low Apgar scores or whose guardians refused consent were excluded from the study. The inclusion of both inborn and outborn cases allowed for a comprehensive analysis of disparities in care and outcomes.

Data collection

**Figure 1:**
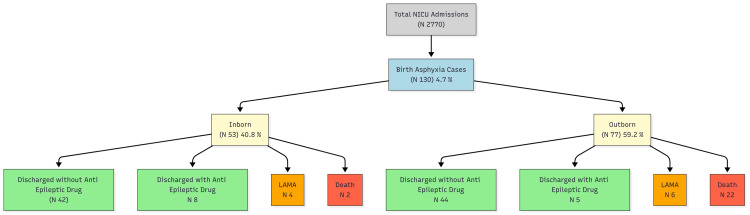
Flowchart of study population Of 2770 NICU admissions, 130 neonates with birth asphyxia were included in the study. The figure illustrates the distribution into inborn and outborn categories, with corresponding clinical outcomes. LAMA: Leave Against Medical Advice

Detailed maternal and neonatal data were collected using structured questionnaires and verified medical records (Appendices). Maternal parameters included age, socioeconomic status, comorbidities, obstetric history, and booking status. Neonatal parameters encompassed birth weight, gender, mode of delivery, complications during NICU stay, and resuscitation requirements. Outcome measures included survival rates, incidence of neurological complications, and duration of hospitalization. Data collection was performed by trained researchers to ensure accuracy and consistency [7]. The study flow is depicted in Figure 1.

Sample size and statistical analysis

The sample size of 130 was determined with reference to previously published epidemiologic studies on the incidence of birth asphyxia and neonatal outcomes [1,7]. Data were analyzed using IBM SPSS (version 22; IBM Corp., Armonk, NY, US). Descriptive statistics summarized the data, while inferential statistics, such as chi-square and t-tests, identified significant associations. A p-value of <0.05 was considered statistically significant. Results were presented through tables, graphs, and descriptive narratives for clarity. Advanced statistical methods were employed to assess the impact of various factors on neonatal outcomes, enabling a nuanced understanding of the data [8].

## Results

Incidence and demographics

Of the 2,770 NICU admissions during the study period, 130 neonates (4.7%) were diagnosed with birth asphyxia, highlighting its significant contribution to neonatal morbidity and mortality [9]. Among these, the majority were male (60.8%), with a mean birth weight of 2.8±0.4 kg. The mean age of mothers was 23.9±4.6 years, with most falling within the 18-24 age range (60%). Maternal hemoglobin levels averaged 9.3±1.4 g/dL, reflecting the high prevalence of anemia among the study population. Baseline maternal and neonatal characteristics are summarized in Table 1.

**Table 1:** Baseline maternal and neonatal characteristics of the study population Data are presented as the number of cases (N) with percentage (%) for categorical variables and mean ± standard deviation (SD) for continuous variables.

Characteristic	Category	N (%) / Mean ± SD
Maternal Age (years)	Mean ± SD	23.9 ± 4.6
	18–24	78 (60.0%)
	25–29	33 (25.4%)
	30–34	17 (13.1%)
	≥35	2 (1.5%)
Maternal Education	Illiterate	8 (6.2%)
	Primary schooling	50 (38.5%)
	Secondary schooling	47 (36.2%)
	Higher secondary	11 (8.5%)
Socioeconomic Status	Lower	103 (79.2%)
	Upper-lower	19 (14.6%)
	Middle	8 (6.2%)
Maternal Hemoglobin (gm%)	Mean ± SD	9.3 ± 1.4
	<7 (Severe anemia)	5 (3.8%)
	7–10 (Moderate)	86 (66.2%)
	10.1–13 (Mild/Normal)	39 (30.0%)
Mode of Delivery	Vaginal	89 (68.5%)
	Assisted vaginal	8 (6.1%)
	Cesarean section	33 (25.4%)
Neonatal Sex	Male	79 (60.8%)
	Female	51 (39.2%)

Maternal risk factors

Anemia was observed in a significant proportion (83.1%) of mothers, emphasizing the need for improved antenatal nutritional care and early intervention programs [10]. Hypertensive disorders were noted in 19.2% of cases, underscoring the importance of maternal health monitoring and management during pregnancy [11]. Prolonged labor complications were observed in 32.3% of cases, indicating the need for enhanced intrapartum care and timely medical interventions [12]. Most mothers (79.2%) belonged to the lower socioeconomic class, highlighting disparities in access to quality care and the role of social determinants in health outcomes [13]. The distribution of maternal risk factors is shown in Table 2.

**Table 2:** Maternal risk factors associated with birth asphyxia Data are presented as number of cases (N) and percentage (%). Percentages are calculated based on the total study population (N=130).

Risk Factor	Number of Cases (n)	Percentage (%)
Anemia (Hb < 10 g/dL)	108	83.1
Hypertension	25	19.2
Prolonged Labor	42	32.3
Antepartum Hemorrhage	12	9.2
Lower Socioeconomic Status	103	79.2

Complications

Hypoxic-ischemic encephalopathy (HIE) was observed in varying severities, with Grade I in 33.1% of cases, Grade II in 28.4%, and Grade III in 10.8% of cases. HIE accounted for a significant portion of neonatal morbidity, necessitating early detection and intervention strategies to mitigate long-term complications [14]. Seizures were recorded in 36.9% of neonates, indicative of severe neurological impairment and necessitating prompt management [15]. Mechanical ventilation was required in approximately 23.1% of neonates, reflecting the critical care needs of this cohort and emphasizing the importance of well-equipped NICUs [16]. The overall mortality rate was 18.5%, with outborn neonates experiencing significantly higher mortality (28.6%) compared to inborn neonates (3.8%). These disparities underscore the critical need for robust referral and transport systems [17]. The major complications observed among neonates with birth asphyxia are presented in Table 3. Comparison of mortality and outcomes between inborn and outborn neonates is detailed in Table 4. Clinical outcomes in neonates with and without HIE are compared in Table 5.

**Table 3:** Neonatal complications in birth asphyxia cases Data are presented as number of cases (N) and percentage (%). Percentages are calculated based on the total number of neonates with birth asphyxia (N=130).

Complication	Number of Cases (n)	Percentage (%)
Hypoxic-Ischemic Encephalopathy (HIE)		
- Grade I	43	33.1
- Grade II	37	28.4
- Grade III	14	10.8
Seizures	48	36.9
Multi-Organ Dysfunction	21	16.2
Requirement for Mechanical Ventilation	30	23.1

**Table 4:** Mortality and outcome comparison between inborn and outborn neonates Data are presented as the number of cases (N) with percentage (%) for categorical variables and mean ± standard deviation (SD) for continuous variables. The chi-square test was used for categorical comparisons, and the independent t-test for continuous variables. Test statistic values (χ² or t) are shown in the second last column. A p-value of <0.05 was considered statistically significant.

Outcome	Inborn (n=52)	Outborn (n=78)	Test Statistic	p-value
Mortality	2 (3.8%)	22 (28.6%)	χ²=12.7	<0.001
Length of NICU Stay (days)	10.4 ± 3.2	15.8 ± 4.7	t=6.1	<0.01
HIE (All Grades)	25 (48.1%)	69 (88.5%)	χ²=28.9	<0.001

**Table 5:** Clinical outcomes in neonates with and without HIE Data are presented as the number of cases (N) with percentage (%) for categorical variables and mean ± standard deviation (SD) for continuous variables. The chi-square test was applied for categorical outcomes, and the independent t-test was used for continuous variables. Test statistic values (χ² or t) are shown in the second last column. A p-value <0.05 was considered statistically significant. HIE - Hypoxic-Ischemic Encephalopathy

Outcome	With HIE (n=94)	Without HIE (n=36)	Test Statistic	p-value
Seizures	41 (43.6%)	7 (19.4%)	χ²=5.53	0.019
Mechanical Ventilation	28 (29.8%)	2 (5.6%)	χ²=7.30	0.007
Multi-Organ Dysfunction	19 (20.2%)	2 (5.6%)	χ²=3.12	0.077
Mortality	21 (22.3%)	3 (8.3%)	χ²=2.53	0.112
Length of NICU Stay (days)	16.5 ± 5.2	9.3 ± 3.8	t=8.68	<0.001

## Discussion

In our cohort, the incidence of birth asphyxia among NICU admissions was 4.7%. This figure is within the range reported from tertiary centers in South Asia, though incidence estimates vary by case definition and setting. Population-level and hospital-based studies have reported higher and lower figures depending on inclusion criteria and the denominator used. Several regional studies have similarly highlighted a higher burden of asphyxia among outborn infants and those with delayed referral, consistent with our observation of worse outcomes in outborn neonates.

Maternal anemia was highly prevalent in our study (108/130; 83.1%). This is substantially higher than national estimates of anemia among pregnant women reported in the National Family Health Survey (NFHS-5), but not unexpected in hospital-based high-risk samples. The NFHS-5 documents a persistently high prevalence of anemia among women of reproductive age and pregnant women in India [18]. Maternal anemia reduces oxygen-carrying capacity and has been associated with adverse perinatal outcomes, including low birth weight, prematurity, and increased risk of perinatal asphyxia in observational studies [19]. These data support the biologic plausibility that high maternal anemia in our cohort contributed to a higher risk of birth asphyxia and perinatal complications.

HIE was a major driver of neonatal morbidity in our series, and neonates with HIE had significantly higher rates of seizures, mechanical ventilation, and prolonged NICU stay. The role of targeted neuroprotective therapies for HIE is well-established: randomized trials and landmark reports, such as the NEJM whole-body hypothermia trial, demonstrated reduced death or disability in term infants with moderate to severe HIE, and hypothermia is now the standard of care where resources allow [20]. Our findings underscore the importance of early recognition of infants at risk of HIE and timely transfer to centers capable of providing neuroprotective care, including therapeutic hypothermia when indicated.

From a health-systems perspective, global agencies and national programs prioritize interventions to reduce newborn mortality attributable to birth asphyxia. WHO and UNICEF emphasize strengthening intrapartum care (skilled birth attendance, fetal monitoring), immediate newborn care (thermal care, timely resuscitation), and functioning referral systems to reduce asphyxia-related deaths [21,22]. Our results, particularly the poorer outcomes among outborn infants and the high prevalence of maternal anemia, point to two actionable areas in our region: improving antenatal optimization (iron/folate programs, screening, and treatment of maternal comorbidities) and strengthening intrapartum monitoring and early referral pathways so neonates at risk receive rapid transport to tertiary NICU care.

In summary, the combination of a high burden of maternal anemia and the predominance of outborn deliveries with delayed referral likely contributed to the frequency and severity of birth asphyxia and HIE in our cohort. Interventions that improve maternal health and intrapartum care, coupled with early identification and transfer of high-risk neonates, may reduce the burden of asphyxia and improve outcomes in similar tertiary-referral settings.

This study has certain limitations. First, it was conducted at a single tertiary care center with a relatively small sample size, which may limit the generalizability of the findings. Second, long-term neurodevelopmental outcomes of the neonates were not assessed, and therefore, the study does not capture the full spectrum of sequelae associated with birth asphyxia. Third, socioeconomic status was classified based on available clinical records rather than standardized indices, which may have introduced classification bias. Finally, resource constraints precluded the use of advanced diagnostic modalities and follow-up assessments. Future multi-center studies with larger cohorts, standardized assessment of socioeconomic determinants, and long-term follow-up are recommended to validate and extend these findings.

## Conclusions

Globally, birth asphyxia continues to be recognized as a major cause of neonatal morbidity and mortality, particularly in low- and middle-income countries. Our findings highlight the need for strengthened antenatal care, timely intrapartum monitoring, and efficient referral systems to tertiary centers. Targeted strategies to address maternal risk factors and ensure early neonatal intervention may substantially reduce the burden of hypoxic-ischemic encephalopathy and improve survival outcomes in similar resource-limited settings.

## Appendices

**Table 6:** Study proforma (data collection tool) Variables recorded in the study proforma. Data were collected using structured clinical records and categorized as demographic, maternal risk factors, intrapartum factors, neonatal variables, and outcomes.

Variable	Category	Response Options
Maternal Age	Demographic	Years (continuous)
Residence	Demographic	Urban / Rural
Socioeconomic Status	Demographic	Lower / Upper-lower / Middle (per modified Kuppuswamy)
Maternal Education	Demographic	Illiterate / Primary / Secondary / Higher
Maternal Hemoglobin	Maternal Risk Factor	g/dL (continuous); categorized as Severe <7, Moderate 7–10, Mild/Normal >10
Maternal Comorbidities	Maternal Risk Factor	Hypertension / Diabetes / Heart disease / Others
Antenatal Care Visits	Maternal Risk Factor	Yes / No (number if applicable)
Labor Characteristics	Intrapartum Factor	Prolonged labor / Obstructed labor / Meconium-stained liquor / Normal
Mode of Delivery	Intrapartum Factor	Vaginal / Assisted vaginal (forceps, ventouse) / Cesarean (GA or spinal)
Place of Delivery	Intrapartum Factor	Hospital / Home / Referred from outside facility
Neonatal Sex	Neonatal Variable	Male / Female
Birth Weight	Neonatal Variable	kg (continuous); categories: LBW <2.5, 2.5–3.5, >3.5
Gestational Age	Neonatal Variable	Preterm / Term / Post-term
APGAR at 1 and 5 minutes	Neonatal Variable	Numeric score
HIE Grading (Sarnat)	Clinical Assessment	Grade I / Grade II / Grade III
Complications	Clinical Outcome	Seizures / MODS / Respiratory failure / Others
NICU Stay	Clinical Outcome	Days (continuous)
Outcome at Discharge	Clinical Outcome	Discharged without AE / Discharged with AE / LAMA / Death
